# Estimating Chronological Age From the Electrical Activity of the Brain: How EEG‐Age Can Be Used as a Marker of General Brain Functioning

**DOI:** 10.1111/psyp.70033

**Published:** 2025-03-16

**Authors:** Thomas M. James, Adrian P. Burgess

**Affiliations:** ^1^ Aston Research Centre for Health in Ageing (ARCHA) and Aston Institute of Health and Neurodevelopment (IHN), College of Health and Life Sciences (HLS), School of Psychology Aston University Birmingham UK; ^2^ ARCHA, HLS, School of Psychology Aston University Birmingham UK

**Keywords:** aging brain, brain integrity, dementia, EEG power Spectrum, human lifespan, partial least squares regression, peak alpha frequency, resting state electroencephalography

## Abstract

With an aging global population, the number of older adults with age‐related changes in the brain, including dementia, will continue to increase unless we can make progress in the early detection and treatment of such conditions. There is extensive literature on the effects of aging on the EEG, particularly a decline in the Peak Alpha Frequency (PAF), but here, in a reversal of convention, we used the EEG power‐frequency spectrum to estimate chronological age. The motivation for this approach was that an individual's brain age might act as a proxy for their general brain functioning, whereby a discrepancy between chronological age and EEG age could prove clinically informative by implicating deleterious conditions. With a sample of sixty healthy adults, whose ages ranged from 20 to 78 years, and using multivariate methods to analyze the broad EEG spectrum (0.1–45 Hz), strong positive correlations between chronological age and EEG age emerged. Furthermore, EEG age was a more accurate estimate and accounted for more variance in chronological age than well‐established PAF‐based estimates of age, indicating that EEG age could be a more comprehensive measure of general brain functioning. We conclude that EEG age could become a biomarker for neural and cognitive integrity.

## Impact Statement

Our goal was to use the EEG power‐frequency spectrum to estimate ‘brain age’. Using multivariate methods to analyze the broad EEG spectrum (0.1–45 Hz), EEG‐age and chronological age were correlated strongly, and EEG‐age was a more accurate estimate and accounted for more variance in chronological age than well‐established Peak Alpha Frequency‐based estimates of age. We conclude that EEG‐age could become a biomarker for neural and cognitive integrity.

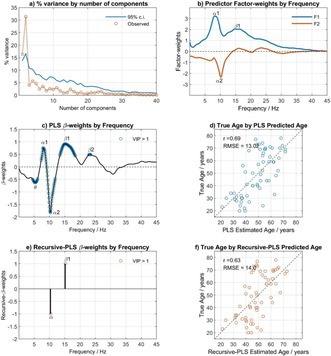

## 1 Introduction

EEG is regarded as a potential candidate for allowing early detection of age‐related conditions such as dementia, thus facilitating better treatment outcomes (Dauwels et al. 2010; Koenig et al. 2020; Poil et al. 2013). When humans sit at rest with their eyes closed, the EEG power spectrum is distributed such that the power at each frequency is approximately proportional to the reciprocal of the frequency $\left(\text{Power}\propto 1/f\right)$ plus a peak in power in the alpha frequency band (He 2014; Lopes da Silva 2013; Pritchard 1992). There is extensive literature on the effects of aging on the EEG, but here, in a reversal of convention, we wanted to use the EEG, both the broad power spectrum (EEG‐age) and the frequency of the peak in power (PAF‐age), to estimate chronological age. EEG‐age and PAF‐age are distinct metrics but can be compared in their ability to estimate chronological age, with the fact that they are distinct as a reason to make the comparison. The applied motivation for this approach was that an individual's brain age might act as a proxy of their general brain functioning, whereby a discrepancy between chronological age and EEG‐estimated age could prove clinically informative by implicating deleterious conditions.

The frequency of the peak in power (i.e., Peak Alpha Frequency, PAF) increases rapidly through childhood, reaching 10 to 11 Hz in early adulthood, before gradually declining to an average of 8–9 Hz by 80 years of age. The change in mean PAF across the human lifespan is arguably the best‐established EEG correlate of age and has been reported not only from the visual inspection of relatively small numbers of EEG recordings (Duffy et al. 1984; Dustman et al. 1993; Hughes and Cayaffa 1977; Mizukami and Katada 2018; Obrist 1954; Stroganova et al. 1999) but also in multiple studies using large sample sizes and automated algorithms (Aurlien et al. 2004; Chiang et al. 2011; Finley et al. 2022; Hashemi et al. 2016; John et al. 1980; Lodder and van Putten 2011; Merkin et al. 2023; Samson‐Dollfus and Goldberg 1979). PAF's short‐term test–retest reliability (e.g., between days, weeks, and months) has been reported as good‐to‐excellent in adults (Joffe et al. 2021; Näpflin et al. 2007; Popov et al. 2023; Salinsky et al. 1991), suggesting that PAF is a stable electrophysiological measure across short intervals in adulthood. Longitudinal studies further confirmed the rapid increase in PAF from the earliest months of life through childhood and adolescence (Cragg et al. 2011; Freschl et al. 2022; Lindsley 1939; Schaworonkow and Voytek 2021; Soroko et al. 2014) before a steady, linear decline in PAF from early adulthood onwards (Kondacs and Szabó 1999). However, longitudinal studies of PAF across adulthood are less common and typically follow participants for only a few years. In summary, although we cannot be sure what any individual's PAF trajectory may be, we can be quite confident of how the population‐level PAF changes with age.

Brain‐aging phenomena are often associated with changes in cognitive functioning, and PAF is no exception. PAF remains positively correlated with scores on a range of tests of cognitive abilities (e.g., attention, processing speed, and memory) even when samples are age‐matched, such that lower PAF predicts worse performance and may reflect inferior information processing (Angelakis et al. 2004a, 2004b; Bornkessel et al. 2004; Clark et al. 2004; Rathee et al. 2020; Surwillo 1961; Trammell et al. 2017). Gray and white brain matter both deteriorate with advancing age (Grady 2012; Hofmann et al. 2022), and a tranche of recent neuroanatomical evidence suggests that changes in the structure of white matter, particularly of thalamocortical networks, play a crucial role in both the slowing of PAF and age‐related decline in cognitive functioning (Bells et al. 2017; Hindriks et al. 2015; Hughes and Crunelli 2005; Minami et al. 2020; Valdés‐Hernández et al. 2010). Understandably then, PAF is lower in persons with age‐related conditions such as mild cognitive impairment (Babiloni et al. 2011; Jelic et al. 2000; Prichep et al. 2006) and dementia (Babiloni et al. 2021; Klimesch et al. 1990; Neto et al. 2015; Penttilä et al. 1985). It has been suggested that PAF may be an electrophysiological marker of general intellectual capacity, ‘g’ (Grandy et al. 2013), or that it reflects specific aspects of cognition such as processing speed (Ociepka et al. 2022). What is certain, though, is that the EEG power spectrum is a marker of the mass‐synchronized action of cortical neurons (Lopes da Silva 2013). This means PAF could provide insights into deleterious age‐related conditions from a general, system‐level perspective (Koenig et al. 2020) that would make it a promising candidate as a biomarker of neural and cognitive integrity.

Over the past 25 years, many neurocognitive and neurobiological theories of aging have emerged to explain age‐related changes in neural and cognitive integrity (Ebaid and Crewther 2020; Grady 2012; McDonough et al. 2022). Two distinct theoretical frameworks persist in deficit versus benefit models. The deficit framework considers dedifferentiation, which comprises diminishing distinctiveness and selectivity of cortical and cognitive processing with increasing age (Koen and Rugg 2019), and noise, which comprises decreasing signal‐to‐noise ratio (SNR) and processing efficiency with increasing age (Cremer and Zeef 1987; Voytek et al. 2015). The benefit framework considers compensation, reserve, and maintenance (Cabeza et al. 2018; Stern 2012), all of which can mitigate age‐related declines in task performance. Compensation is the recruitment of neural and cognitive resources to meet task demands, with the levels of compensation often proportional to chronological age. Reserve is the presence of neural and cognitive resources, and maintenance is the preservation of those resources. Deficit and benefit models are still being refined based on new evidence (Aron et al. 2022; Pichot et al. 2022), but they continue to acknowledge a changed, often wider distribution of cortical processing across the adult lifespan. This change can go some way to explaining the slowing of PAF because larger networks often synchronize at lower frequencies (Lopes da Silva 2013; von Stein and Sarnthein 2000), which means that PAF may reflect the scale of cortical processing, and this interpretation adheres to the system‐level interpretation of neural and cognitive integrity found in other EEG literature (Koenig et al. 2020). In summary, a decline in PAF is an oft‐reported, normal brain‐aging phenomenon that likely reflects changes in neural and cognitive integrity, although no specific mechanism or interpretation has been established beyond reasonable doubt. Any relationship between age, neural function, and cognition is likely to be complex and masked by numerous factors, such as those outlined by the deficit vs. benefit models, and a general approach to integrity is recommended.

Currently, there are practical limitations in using PAF as a proxy to track age‐related changes in integrity. One limitation is that the frequency resolution of a typical EEG power spectrum is poor. A widely used method to estimate the EEG power spectrum, Welch's method (Welch 1967), involves splitting an EEG recording into short, stationary, artifact‐free segments, performing the Fast Fourier Transform (FFT) on each segment, and averaging. The frequency parameters depend on the sampling rate of the EEG recording and the length of the segments, which, given typical values (e.g., 500 Hz and 4 s), will give a frequency resolution no better than about 0.25 Hz. With the average decline in PAF estimated at around 1 to 2 Hz over six decades, a frequency resolution of 0.25 Hz is sufficient to detect relatively large changes in PAF across the lifespan. Alternative methods of estimating the EEG power spectrum, such as autoregressive methods (AMs), are rarely used even though they offer the advantages of higher frequency resolution and smoother EEG power spectra (Gersch 1970). AMs estimate the EEG power spectrum by regressing each value of the EEG time series onto a set number of past values. This number, called the model order, determines the period of the lowest frequency detectable, and a higher model order allows for better frequency resolution (Takalo et al. 2005). As a statistical approach, AMs model error in the data, which enables them to produce smoother EEG spectra than conventional methods, and this should improve the ability to interrogate and track fine‐grained changes in PAF.

Another limitation in using PAF to track age‐related changes is that there is no consensus as to the best way to estimate PAF, because, as Corcoran et al. (2018) suggest, defining PAF “poses a nontrivial challenge, and may rely on subjective assessments or arbitrary criteria” (p. 2). In other words, there is no ‘true’ PAF, but there is a set of well‐established, distinct approaches to estimating PAF. A researcher who defines PAF as the frequency of maximum power in the EEG spectrogram (i.e., the Naïve Peak Alpha Frequency method, N‐PAF) is faced with several problems. One is that the N‐PAF method offers no guidance on how to handle cases where there may be two or more peaks in the alpha frequency range, nor where there are no peaks at all (Chiang et al. 2008; Olejarczyk et al. 2017). Another problem is that PAF varies across scalp sites (Mahjoory et al. 2020; Quinn et al. 2021), although the extent of this topographical variation and its relationship to the SNR in the EEG spectrogram remains undetermined. One way to deal with within‐subject variation is by calculating the arithmetic mean PAF, which requires taking an average of the estimates of PAF obtained from different scalp locations or calculating a single estimate of PAF from the EEG power spectrum averaged across scalp locations. A third issue is that the N‐PAF method assumes fixed frequency bounds (e.g., 7.5 to 12.5 Hz for alpha frequency), which is questionable given that PAF varies with age, and mis‐defined boundaries can cause problems due to the exclusion of alpha peaks or inclusion of peaks that are not alpha (Donoghue et al. 2020, 2021; Haegens et al. 2014). A complete method for estimating PAF should be able to readily deal with any number of peaks, within‐subject variance, and individual differences in the alpha frequency band. No existing method meets all these criteria, but several other demonstrably useful ways of estimating PAF do exist.

These include the Corcoran‐PAF (C‐PAF) method, proposed by Corcoran et al. (2018), which involves identifying the PAF from the smoothed EEG $\log\left(\text{power}\right)$ spectra across multiple channels, taking the average weighted by channels' SNR. The Klimesch‐PAF (K‐PAF) method, first proposed by Klimesch et al. (1990, 1993) and adapted by Corcoran et al. (2018), is based on the center‐of‐gravity of the individually defined alpha band. Corcoran et al. (2018) have shown that both the K‐PAF and C‐PAF methods can estimate PAF in nearly all participants. Furthermore, in simulations where there was a single peak, both methods outperformed N‐PAF, and C‐PAF was superior to K‐PAF in cases of low SNR. The Modeled‐PAF (M‐PAF) method is a curve‐fitting approach to estimating PAF, previously used as an automatic algorithm in large‐scale studies. It involves modeling the EEG power spectrum as one or more frequency bands (typically assumed to follow a Gaussian distribution) superimposed on a 1/f slope (Chiang et al. 2011; Lodder and van Putten 2011). The mean frequency of the Gaussian distribution used to represent the alpha frequency is taken as the estimate of PAF.

The N‐PAF, K‐PAF, C‐PAF, and M‐PAF methods are not free from assumptions, and each method requires some user input. For example, C‐PAF requires pre‐defined alpha bounds and a minimum value for the power of alpha peaks. M‐PAF involves fitting a non‐linear model, and solving the model requires the user to pre‐define boundaries for the parameters. These parameters include the upper and lower limits of the mean and standard deviation of the Gaussian that represents the alpha frequency band. Once the parameters are set, though, PAF can be estimated without further user intervention. One advantage of M‐PAF is that it can be readily extended to frequency bands other than alpha and incorporates the 1/f slope; thus, both periodic (e.g., traditional oscillatory bands) and aperiodic (e.g., 1/f slope) components of the EEG power spectrum can be considered (Donoghue et al. 2020). That said, peaks in other frequency bands are rarely clear and, thus, rarely reported, and peaks that do occur tend to be broader than in alpha. Consequently, age‐related changes across the EEG power spectrum are traditionally examined via changes in oscillatory power in pre‐defined frequency bands, but the results vary widely and depend on the methodology and statistical approach used by the researchers (Cragg et al. 2011; Dauwels et al. 2010; Duffy et al. 1993; Gómez et al. 2013; Ishii et al. 2017; Vysata et al. 2012). For example, studies that account for age‐related changes in the 1/f slope concurrently with oscillatory power have produced contrasting evidence to the traditional approaches, although the findings have not been entirely consistent (Donoghue et al. 2021; Finley et al. 2022; Merkin et al. 2023; Schaworonkow and Voytek 2021; Trondle et al. 2022).

One way to circumvent the difficult choices required when estimating PAF is to consider a distinct metric that, to our knowledge, has not previously been used to estimate chronological age but could provide a robust assessment. That metric is age‐related changes across the broad EEG power spectrum (EEG‐age) using multivariate methods such as Partial Least Squares (PLS) regression. PLS, which can be interpreted as a combination of principal component analysis and regression, is particularly useful where the independent predictor variables are highly correlated, as they invariably are with spectral data. Widely used for analyzing spectral data in Chemistry (Wold et al. 2001), PLS has been used with EEG data too, particularly event‐related potential data (Lobaugh et al. 2001; McIntosh and Lobaugh 2004), with multiple tutorials now available on how to run PLS‐based analyses with different types of neuroimaging data (e.g., Alin et al. 2009; Krishnan et al. 2011). PLS is used when independent predictor variables can be represented as a two‐dimensional matrix (e.g., participant‐by‐spectra); however, PLS can be generalized to cases with n‐dimensional independent variables, where n is any integer, via Multilinear Partial Least Squares (M‐PLS) regression (Bro 1997; Bro and Kiers 2003). This makes it possible, for example, to analyze the relationship between spectral data from multiple participants and channels (participant‐by‐channel‐by‐spectra) and age in the same analysis. To our knowledge, neither PLS nor M‐PLS has previously been used to examine the broad EEG power spectra (e.g., 0.1 to 45 Hz) with the focused aim of estimating chronological age, yet multivariate approaches have advantages that may provide more robust results than the more conventional approaches when attempting to match chronological age with brain age. For example, multivariate analyses can use all information in the EEG spectra dataset at the same time, work when there are no visible peaks in the spectra, and reduce subjective user input.

If it were possible to reliably estimate an individual's chronological age based on their broad EEG power spectrum, then this EEG‐age could be compared with actual chronological age and any discrepancy between the two might reflect some age‐related condition, either protective or deleterious. For example, using good normative data, the number of standard deviations above or below the mean EEG‐age for the person's chronological age could be a useful biomarker for mild cognitive impairment or early dementia. Developing neuroimaging biomarkers is not a particularly novel venture (Dauwels et al. 2010; Franke and Gaser 2019; Hofmann et al. 2022; Koenig et al. 2020; Poil et al. 2013; Snaedal et al. 2012) and follows in the traditions of Roy John's Neurometrics (John et al. 1977). Furthermore, several attempts have been made to operationalize an EEG‐age concept in all but name by using machine learning to identify age‐related electrophysiological components from a plethora of popular EEG metrics (e.g., Al Zoubi et al. 2018; Sun et al. 2019). However, we wanted to take a more targeted approach, using alternative EEG data analyses that may provide more robust results to estimate our version of EEG‐age in a sample of healthy adults from across the chronological age range. This approach to using EEG‐age is like that pioneered by Miloš Matoušek, Ingemar Petersén, and Jiri Wackermann (Matoušek and Petersén 1973; Wackermann and Matoušek 1998). However, we also wanted to incorporate a more age‐balanced sample of exclusively healthy adults and use more sophisticated EEG procedures and analyses that can inspect frequency and amplitude separately as qualitatively distinct aspects of EEG power spectra. We also wanted to compare proxy measures of general cognitive integrity alongside EEG‐estimated age to probe age‐related processes (e.g., dedifferentiation and noise versus reserve) from a system‐level perspective.

Our objective was to use the EEG power‐frequency spectrum to estimate chronological age as EEG‐age. First, we wanted to compare the well‐established variants of PAF on their ability to estimate age. Based on previous evidence, we predicted that PAF would be negatively correlated with chronological age, and we aimed to determine which estimate of PAF (M‐PAF, C‐PAF, K‐PAF, or a variant of N‐PAF) correlated most strongly and provided the most accurate estimate of chronological age. Second, we sought to explore the potential of multivariate analyses of the broad EEG spectrum (0.1 to 45 Hz), using PLS and M‐PLS as qualitatively distinct analytical approaches to PAF that have not previously been used to estimate chronological age. Third, we wanted to examine the relationships of PAF age and PLS EEG‐age with already established proxy measures of general cognitive integrity.

## 2 Methods

### 2.1 Participants

Sixty healthy adults (24 men, 36 women; 5 left‐handed, 55 right‐handed) volunteered to participate, which allowed for 10 participants per decade of chronological age across six decades (M = 49 years, SD = 17.9, Range = 20–78). Having reached our resource limit, a sensitivity power analysis for a univariate negative correlation between PAF and age using Pearson's *r*, with an alpha of 0.05 and a beta of 0.2 [0.1], calculated a minimum detectable effect size of −0.31 [−0.37] (calculated using G*Power, version 3.1.9.7; Faul et al. 2009). Participants had a mean of 17 years in formal education (SD = 4.0, Range = 7–27). Fifty‐two participants identified as White, seven as Asian, and one as Black, with all participants recruited via Aston University's advertising portals, which include the ARCHA Panel that comprises older adults from around the UK who volunteer to take part in studies at Aston University. This study received a favorable opinion from Aston University's Research Ethics Committee and was carried out in accordance with the Declaration of Helsinki. Written informed consent was obtained from each participant, and they were reimbursed £15 for their participation.

All participants actively reported having no experience of traumatic brain injury, no diagnosis of neurological or psychiatric disorder, and no known cognitive impairment. Participants were screened for depression via the Geriatric Depression Scale‐15 (GDS‐15; Sheikh and Yesavage 1986), as severe depression may confound measures of cognition (Byers and Yaffe 2011; Morimoto and Alexopoulos 2013) and PAF (Tement et al. 2016; Zhou et al. 2023). Participants were also screened for cognitive impairment via the Quick Mild Cognitive Impairment Screen (QMCI; O'Caoimh and Molloy 2017). The QMCI is a paper‐based, quick‐to‐administer assessment of general cognitive ability, comprising validated cut‐off scores for normal cognitive functioning, mild cognitive impairment, and dementia (O'Caoimh et al. 2017). We included the QMCI not only as a screening tool, though, where healthy adults should report scores that are isolated to the normal category, but as our estimate of general cognitive integrity, a proxy measure of dedifferentiation and noise.

Aging is associated with more than depression and detrimental changes in neural and cognitive integrity, and there are neurocognitive and neurobiological agents that provide protective benefits, like compensation, reserve, and maintenance. Therefore, participants also completed the National Adult Reading Test (NART; Nelson 1982; Nelson and Willison 1991), which is a well‐established tool that was designed to be robust to early age‐related decline in integrity (normal decline and dementia; Maddrey et al. 1996; Nelson and McKenna 1975; Nelson and O'Connell 1978). Consequently, the NART‐IQ is a good estimate of premorbid intelligence, which is positively correlated with the Wechsler Adult Intelligence Scale's WAIS‐IQ score (a large correlation coefficient of 0.69; Bright et al. 2018) but takes far less time to complete than the WAIS. We converted the raw NART scores to estimates of the Wechsler Adult Intelligence Scale score (WAIS‐IV; Wechsler 2008), called NART‐IQ, via a validated conversion, NARTIQ=126.41−0.9775×NART errors (Bright et al. 2018). Overall, being more reflective of crystallized than fluid intelligence (Bright et al. 2002; Cattell 1963), the NART‐IQ was included as an efficient, proxy measure of mechanisms that support cognitive functioning, particularly reserve (Boyle et al. 2021), in contrast to the QMCI. Years in education is another such proxy measure, albeit with a weaker reported effect size than NART‐IQ (Boyle et al. 2021; Bright et al. 2002).

### 2.2 EEG Recording

DC‐EEG was recorded for 4 min while participants were sitting at rest, on a comfortable chair in a quiet room, with their eyes closed. The EEG amplifier was an ANT eego (EE‐215; input impedance of > 1 GΩs), and the EEG recording software was ANT's eego suite (version 1.7.0) running on an Intel Dell Venue 8 Pro 5855 tablet with a Windows 10 operating system. The sampling rate was 500 Hz, with a 24‐bit resolution and a 0 (DC) to 130 Hz signal bandwidth set by the manufacturer: 0.26×Sampling Rate. Participants wore a 64‐channel ANT Neuro Waveguard Originals EEG cap, with sintered Ag/AgCl electrodes arranged in a 10/10 layout and noise‐shielded cabling. AFz and CPz electrodes were the ground and online reference respectively, set as default for the ANT eego by the manufacturer, but data was re‐referenced to the common average offline before the main analysis. Conductive OneStep Cleargel was applied to each electrode via a blunt syringe.

With a mean impedance of 15.48 kΩs (SD = 13.33, Range = 0.01 to 120.50), most electrodes had similar impedances that were less than the reported optimal cut‐offs of 40 kΩs (Ferree et al. 2001; 94% of 3780 electrodes) and 50 kΩs (Kaneko et al. 2021; 97% of 3780 electrodes) for the high‐impedance ANT system. For one participant, one mastoid electrode (M1) reached 120.50 kΩs, although this was still below the workable maximum of 200 kΩs (Ferree et al. 2001). Four additional electrodes were applied to each participant, allowing for bipolar EOG recordings of vertical (electrodes placed above the right eyebrow and beneath the right eye) and horizontal (electrodes placed on the right and left temple areas) eye movements. This setup remained identical for each participant, with room temperature kept at about 20°C and a low level of ambient light.

### 2.3 Data Preparation

EEG data were pre‐processed and analyzed in MATLAB R2019b. DC‐EEG recordings can contain long slow trends that deviate substantially from zero in the absence of any artifact. Therefore, the raw data was demeaned, and then a time‐varying baseline was used (spanning 1025 data points, ± 2.048 s), derived from a standard 3rd‐order Savitzky–Golay filter (Press and Teukolsky 1990), and raw data that deviated from the Savitzky–Golay filtered signal by ±120 μV were excluded. The largest possible contiguous segment of artifact‐free EEG was selected for each person. Over 100 s of EEG was obtained for each person (M = 205 s, SD = 58, Range = 103 to 343, with one EEG recording that exceeded 4 min) without the need for EOG correction. However, for 12 participants, channels had to be excluded to achieve this target duration and avoid noise contamination of the PAF and spectral estimates, to be left with the most reliable estimates possible per participant using the available data. In most cases, fewer than 5 channels were excluded, except for four cases where 6, 7, 9, and 16 channels, respectively, were excluded. Subsequent agglomeration across channels and/or participants simply used the data that remained. This data preparation was completed without reference to other data, such as chronological age, but we later checked whether age and length of recording were correlated, and they were not (*r* = −0.05, *p* = 0.730).

### 2.4 Signal Analysis

For the N‐PAF and M‐PAF methods, an autoregressive power spectral density estimation was used with the covariance method of model order 256 that covers a span of 512 milliseconds (256×2; MATLAB function ‘pcov.m’), allowing for the estimation of spectral density values from 0.1 to 45 Hz at a resolution of 0.1 Hz compared to around 0.25 Hz for the Welch method. Frequencies above 45 Hz were excluded from analysis because of their low SNR, including the United Kingdom's 50 Hz mains electricity noise, and frequencies below 0.1 Hz were also excluded. To prevent the highest power values at low frequencies from biasing the statistical analyses, the EEG power spectrum was converted to amplitude by taking the square root and then the logarithm (base 2) to create a $\log_{2}\left(\text{amplitude}\right)$ spectrum that was used in subsequent analyses (Burgess 2019). The K‐PAF and C‐PAF methods were implemented using an openly accessible MATLAB code called ‘restingIAF’, which has been shared by Corcoran et al. (2018). For both methods, spectral analysis followed the traditional Welch FFT‐method implemented with a 4096‐millisecond Hamming‐tapered window with 50% overlap. The power spectra were then normalized and smoothed using a 5th‐order Savitzky–Golay filter (spanning 11 frequency bins, ± 2.69 Hz).

The N‐PAF method was implemented first. This approach involved a simple identification of all peaks in the spectrum between 7 to 13 Hz at each channel for each participant. A peak was defined as any part of the spectrum where the gradient of the amplitude $\left(\mathrm{\delta A}/\mathrm{\delta f}\right)$ changed sign. If more than one peak was identified, the N‐PAF was defined as the peak with the highest amplitude. No identifiable peak occurred in < 2% of channels (67 out of 3780 channels), and these channels were each listed as a missing datum. The normal way to calculate a univariate estimate of PAF for the N‐PAF method is to calculate an average across channels. Consequently, channels are weighted equally regardless of their SNR. Alongside the classic N‐PAF method, we also implemented an alternative version of N‐PAF that we named the Direct Estimate‐PAF (D‐PAF). This involved singular value decomposition (SVD; Harner 1990) of the multivariate channel‐by‐spectra matrix. Spectra were the $\log_{2}\left(\text{amplitude}\right)$ values estimated at 0.1 Hz intervals. SVD deconstructs an EEG input into a linear combination of components and, when used to improve the SNR of a univariate PAF estimate by data reduction of the underlying EEG spectra, emphasizes features of the EEG power spectrum that are consistent across channels in an individual. This is comparable to creating a weighted‐mean EEG power spectrum, where the weighting is by the common variance across channels. In the current study, the first extracted component accounted for a large majority of variance in every participant (M = 93.9% variance, SD = 3.2%, Range = 82.2%–98.6%). Post‐SVD, the summary measure of PAF was obtained simply by identifying the peak with the highest amplitude in the 7 to 13 Hz range.

K‐PAF and C‐PAF methods were implemented next, in accordance with the ‘restingIAF’ code that was shared by Corcoran et al. (2018). The K‐PAF approach involved finding the center‐of‐gravity (i.e., amplitude‐weighted mean frequency) in the alpha range, using a smoothed Welch's EEG spectrum. The bounds of the alpha band were defined when the gradient of the amplitude was equal to zero. The summary estimate of PAF was derived from data across all available channels, with each channel contributing equally. The C‐PAF approach also used smoothed EEG spectra, with the peak frequency being defined at each channel (as in N‐PAF) before averaging to produce the summary measure. This averaging involved excluding channels with a low amplitude alpha peak, such that the minimum amplitude of a peak would be a standard deviation above the estimate predicted by a regression model of the log‐transformed power spectral density. To finish, the SNR‐weighted mean of the remaining PAFs was calculated.

Finally, the M‐PAF method was implemented in close alignment with the methods used in previously published papers that correlated M‐PAF with age (e.g., Chiang et al. 2011; Lodder and van Putten 2011). It assumed that the EEG power spectrum consists of an aperiodic 1/*f* slope plus Gaussian distributions representing the peaks of five different periodic frequency bands (i.e., Theta, Alpha, Beta1, Beta2, and Gamma). Specifically, the $\log_{2}\left(\text{amplitude}\right)$ spectrum consisted of a $A_{0}.\frac{1}{f^{m}}+k$ component, where ***f*** is the frequency raised to the power **m**, weighted by a constant, $A_{0}$, with an offset, ***k***, plus the five frequency bands. These bands were represented by a Gaussian distribution, $A_{i}e^{\left[-\frac{1}{2}{\left(\frac{f-\mu_{i}}{{\sigma_{i}}^{2}}\right)}^{2}\right]}$, with maximum amplitude, $\;A_{i},$ mean peak frequency, $\mu_{i}$ and standard deviation, $\sigma_{i}$. Combined this gives:

(1) $$\mathrm{lo}g_{2}A\left(f\right)=A_{0}.\frac{1}{f^{m}}+\sum_{i=1}^{5}A_{i}e^{\left[-\frac{1}{2}{\left(\frac{f-\mu_{i}}{{\sigma_{i}}^{2}}\right)}^{2}\right]}+k$$

**FIGURE 1:**
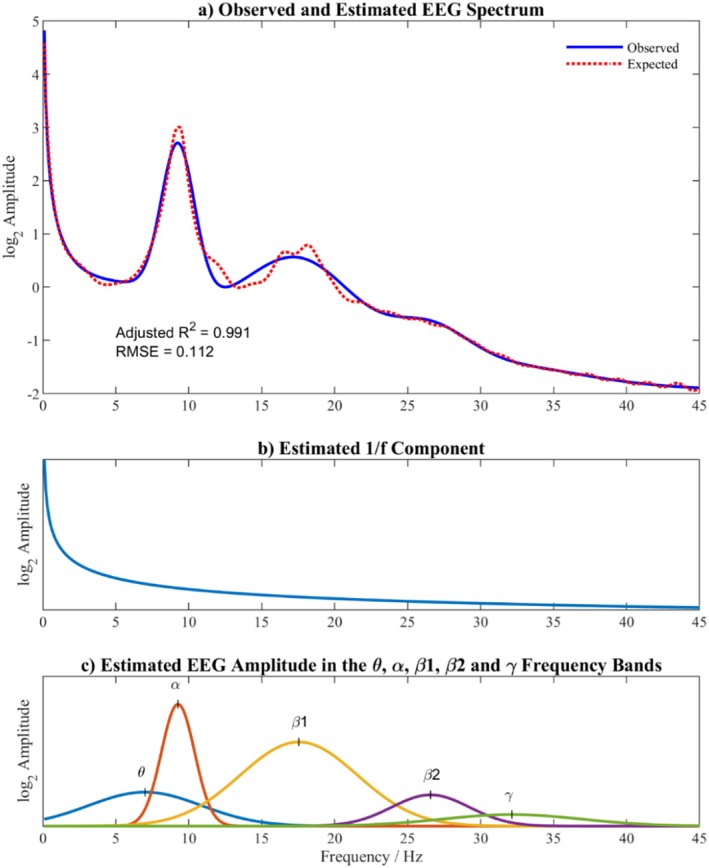
The model EEG density spectrum. (a) shows an individual's observed spectrum compared with the modeled spectrum; (b) shows the 1/f slope; (c) shows the five Gaussian components representing the theta, alpha, beta1, beta2, and gamma frequency bands.

The model was fitted using non‐linear least squares (MATLAB function ‘fit.m’) with conventional constraints: m<0, $0\leq A_{i}\leq 10\;\mathrm{lo}g_{2}\left(\mathrm{\mu V}/\mathrm{Hz}\right)$, peak frequencies, $\mu_{i}$, to be in the ranges 1–7, 7–13, 13–21, 21–29, and 29–47 Hz, and maximum widths, $\sigma_{i}$, to be 15, 10, 30, 30, and 30 Hz for the five frequency bands respectively. An example fit of this model to an individual's EEG power spectrum is shown in Figure 1 (M = 0.995 R^2^_Adjusted_, SD = 0.006, Range = 0.963 to 0.999). The model was not fitted to each channel but, like D‐PAF, to the first component of a SVD, so one summary estimate of PAF accounted for all channels again. An overview of the procedural differences between the PAF methods is shown in Table 1.

**TABLE 1:** Differences between PAF estimation methods.

	Method name	Spectral analysis	Channel treatment	Peak identification
N‐PAF	Naïve‐Peak Alpha Frequency (Chiang et al. 2008)	Autoregressive Method (AM; Gersch 1970)	No averaging	Per channel, the alpha peak with the highest amplitude
D‐PAF	Direct Estimate‐Peak Alpha Frequency	Autoregressive Method (AM; Gersch 1970)	Singular Value Decomposition (SVD; Harner 1990)	The SVD's first‐component's alpha peak with the highest amplitude
M‐PAF	Modeled‐Peak Alpha Frequency (Chiang et al. 2011; Lodder and van Putten 2011)	Autoregressive Method (AM; Gersch 1970)	Singular Value Decomposition (SVD; Harner 1990)	The SVD's first‐component's frequency of the Gaussian distribution used to represent the alpha frequency
C‐PAF	Corcoran‐Peak Alpha Frequency (Corcoran et al. 2018)	Welch‐FFT (Welch 1967)	Weighted‐averaging across smoothed EEG spectra‐PAF	The SNR‐weighted, averaged alpha peak with the highest amplitude
K‐PAF	Klimesch‐Peak Alpha Frequency (Klimesch et al. 1990, 1993)	Welch‐FFT (Welch 1967)	Averaging across smoothed EEG spectra‐PAF with individually defined alpha bands	The amplitude‐weighted, averaged mean frequency

### 2.5 Statistical Analysis

The correlations between chronological age and estimates of premorbid intelligence (i.e., NART‐IQ), cognitive functioning (i.e., QMCI scores), and PAF, as well as between PAF age and PLS EEG‐age respectively with intelligence and functioning, were assessed using the Pearson product–moment correlation coefficient (Pearson's *r*). We used the Glass & Hopkins method (Glass and Hopkins 1996; IBM Support 2020) to statistically compare the strengths of the correlation coefficients. The levels of agreement between each method of estimating PAF were assessed via a Bland–Altman analysis (Bland and Altman 1986), which plots the difference in PAF between two methods' estimates against the average PAF of the same two estimates. The ability of PAF to predict chronological age was assessed using linear regression. Bayesian Hierarchical Regression (BHR) was used to evaluate null models against alternatives in predicting chronological age and cognitive functioning with PAF age and PLS EEG‐age, to judge the levels of evidence in favor of the null (*H*_*0*_) by using established cut‐offs (Jeffreys 1961; Wagenmakers et al. 2011). The default Jeffreys‐Zellner‐Siow (JZS) prior with an r scale of 0.354 was used due to this being the first time, at least to the authors' knowledge, that PLS had been used to estimate EEG‐age.

The ability of the broad EEG power spectrum of the 0.1 to 45 Hz range to predict chronological age (i.e., EEG‐age) was assessed using PLS (MATLAB function ‘plsregress.m’). The predictor was the first component of the SVD of the channel‐by‐spectra matrix. The number of PLS factors to be extracted needed to be defined, which was done via permutation testing. Specifically, the significance of each factor was determined by comparing the amount of variance accounted for by each additional factor with the percentage of variance accounted for by multiple permutations (1000 times) of the age data. Identification of the most age‐responsive components per factor was determined using recursive weighted‐PLS, R‐PLS (Rinnan et al. 2014), which iteratively reweights the variables using the regression coefficients calculated by PLS until only a small number of the most important predictors remain. In this way, age‐predictive weightings for each part of the EEG spectrum are reduced to a small number of critical frequencies that can be used to predict age nearly as accurately as the full spectrum.

PARAFAC is a generalization of Factor Analysis to higher‐order data sets and, when used in combination with PLS, forms M‐PLS. Specifically, we used M‐PLS for the higher‐order array of participants‐by‐channels‐by‐spectra. The objective remains to represent as much of the variance in the complete dataset by a smaller number of factors. PARAFAC's advantage over other data reduction techniques, such as Principal Components Analysis, is that there is a unique solution for each number of factors extracted, and no rotation of the factors is required. We used M‐PLS (MATLAB code N‐way Toolbox version 3.20; Andersson and Bro 2000) to further examine the ability of EEG age to predict chronological age. The number of factors to extract was determined by examining the sum‐squared error, core consistency diagnostic (CORCONDIA; Bro and Kiers 2003), and changes in the number of iterations required to fit the model at each number of factors. Twenty‐five replications were produced for each number of factors, from 1 to 10.

## 3 Results

### 3.1 Correlations Between Chronological Age, NART‐IQ, and Cognitive Performance

The mean NART‐IQ was 115 (SD = 5.8, Range = 102–125), indicating that the sample was generally above average and less varied in premorbid intelligence than the general population. There was also a significant positive correlation between NART‐IQ and chronological age (*r* = 0.54, *p* < 0.001), which reflects the high premorbid intelligence (e.g., reserve) of older adult volunteers on the ARCHA Panel. The mean score of cognitive performance on the QMCI was 77.0 (SD = 7.4, Range 61–93), indicating that the sample had normal cognitive functioning. Two individuals (aged 59 and 78) scored just below the optimal unadjusted cut‐off score for cognitive impairment (< 62). However, when accounting for their chronological age and number of years in education, both participants scored above their optimal adjusted cut‐off score (< 60 and < 54 respectively). Age was not significantly correlated with cognitive performance (*r* = −0.18, *p* > 0.05), and NART‐IQ was not significantly correlated with cognitive performance (*r* = 0.12, *p* > 0.05).

The mean score of mood on the GDS‐15 was 2 (SD = 2.6, Range = 0–11), indicating that the sample was presenting with normal mood on average. Six individuals (aged 24, 24, 32, 38, 52, and 59) scored on or just above the cut‐off score for mild depression (≥ 5), and two individuals (aged 44 and 68) scored on or just above the cut‐off score for moderate depression (≥ 10). No participants scored ≥ 12, indicating no cases of severe depression. Chronological age (*r* = −0.07, *p* > 0.05), cognitive performance (*r* = −0.16, *p* > 0.05), and NART‐IQ (*r* = −0.14, *p* > 0.05) were not significantly correlated with mood. Participants had a mean of 17 years in formal education (SD = 4.0, Range = 7 to 27), and chronological age (*r* = −0.16, *p* > 0.05), cognitive performance (*r* = 0.01, *p* > 0.05), NART‐IQ (*r* = 0.09, *p* > 0.05), and mood (*r* = −0.14, *p* > 0.05) were not significantly correlated with years in formal education.

### 3.2 Topographical Variation of PAF Estimated by the N‐PAF Method

**FIGURE 2:**
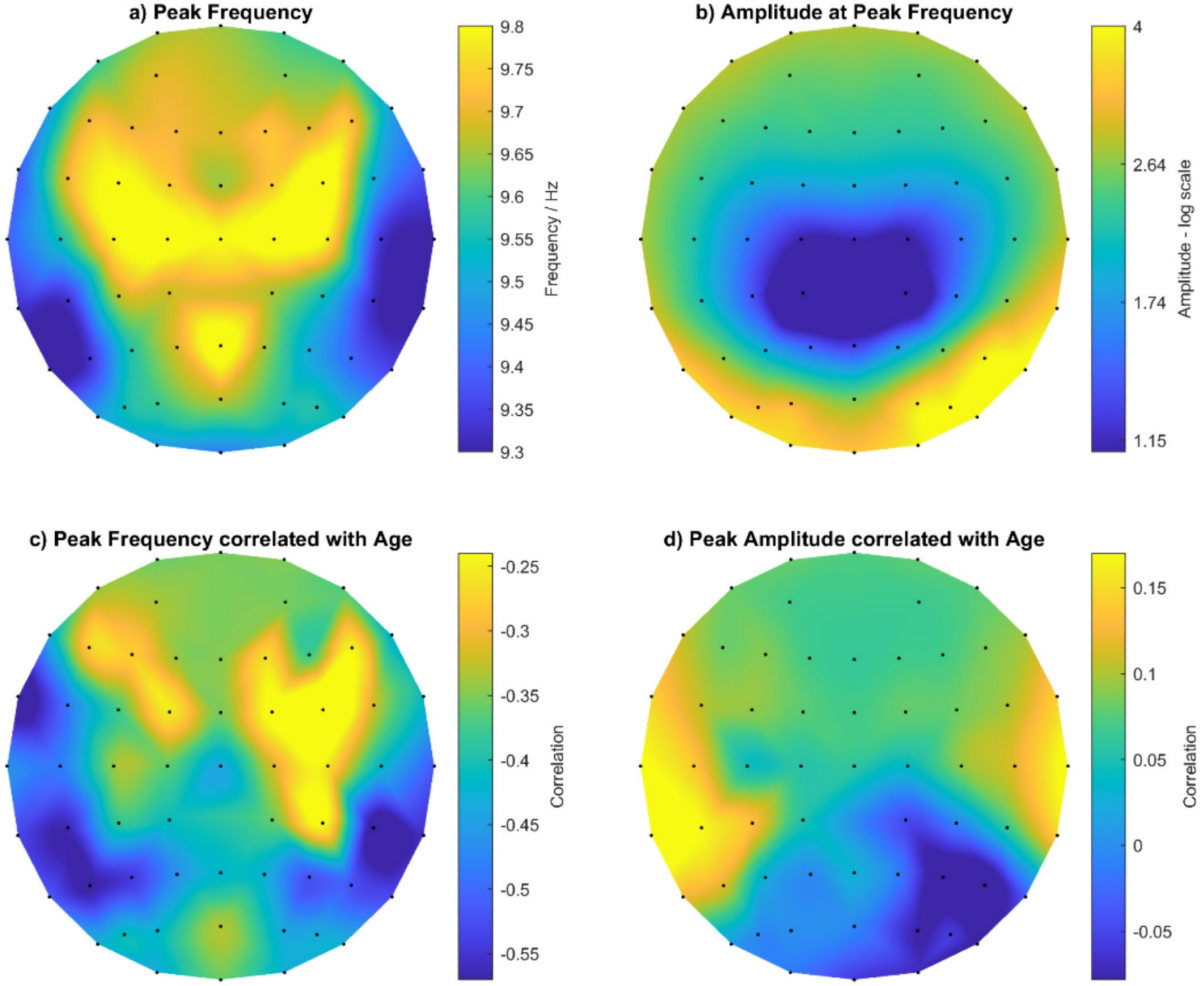
Summary of PAF and amplitude estimated by the N‐PAF method, and their relationships with chronological age. (a) Shows the distribution of PAF, averaged across participants, by topography; (b) shows the distribution of amplitude at each PAF, averaged across participants, by topography; (c) shows the Pearson's *r* correlation coefficients between age and PAF by topography; (d) shows the Pearson's *r* correlation coefficients between age and amplitude at each PAF by topography.

D‐PAF, C‐PAF, K‐PAF, and M‐PAF are methods that aim to summarize PAF across all available channels, so we considered the N‐PAF method in isolation because it best estimates PAF at channels separately. Figure 2a visualizes the topographical distribution of PAF averaged across all participants (M = 9.57 Hz, SD = 0.16, Range = 9.26 to 9.86), with the amplitude at each mean PAF value shown in Figure 2b (M = 1.22 log_2_A, SD = 0.58, Range = −0.58 to 2.15). Maximum power at PAF occurred at right occipital sites for eyes closed at rest. There were significant negative correlations (*p* < 0.05, uncorrected for multiple comparisons) between chronological age and PAF at 52 channels (Figure 2c), indicating that PAF decreases with increasing age across most channels and with little topographical variation (M = 0.40 *r*, SD = 0.01). The largest clusters of negative correlations were at temporoparietal sites, but the maximum correlation coefficient was FT7 (*r* = −0.61, *p* < 0.001). The amplitude at each PAF per channel revealed no significant correlations with age (Figure 2d; maximum correlation coefficient of *r* = 0.25, *p* > 0.05, at TP7). In summary, there was little topographical variation and a consistent correlation between PAF and chronological age.

To formally evaluate the importance of topographical variation on N‐PAF's estimate of PAF, we estimated the proportion of variance attributable to between‐subject (B‐S; participants) and within‐subject (W‐S; channels) factors using the Minimum Norm Quadratic Unbiased Estimator (MINQUE in SPSS Statistics 26.0.0; IBM Corporation 2021; Rao 1972) with uniform random effect priors (i.e., scheme 1). B‐S factors accounted for 71% of the total variance in PAF, and W‐S factors, which represent the consistency of the topographical distribution of PAF across individuals, accounted for 2% of the variance, which leaves 27% attributable to measurement error. In the current study, accounting for only 2% of the total variance in PAF, W‐S variation could be treated as noise (i.e., inconsistent SNR), which is consistent with the little topographical variation shown in Figure 2 and, thus, supports using a single summary value to represent an individual's PAF. The next section will compare four summary measures of PAF, estimated via D‐PAF, M‐PAF, C‐PAF, and K‐PAF methods.

### 3.3 Comparing D‐PAF, M‐PAF, C‐PAF, and K‐PAF

**FIGURE 3:**
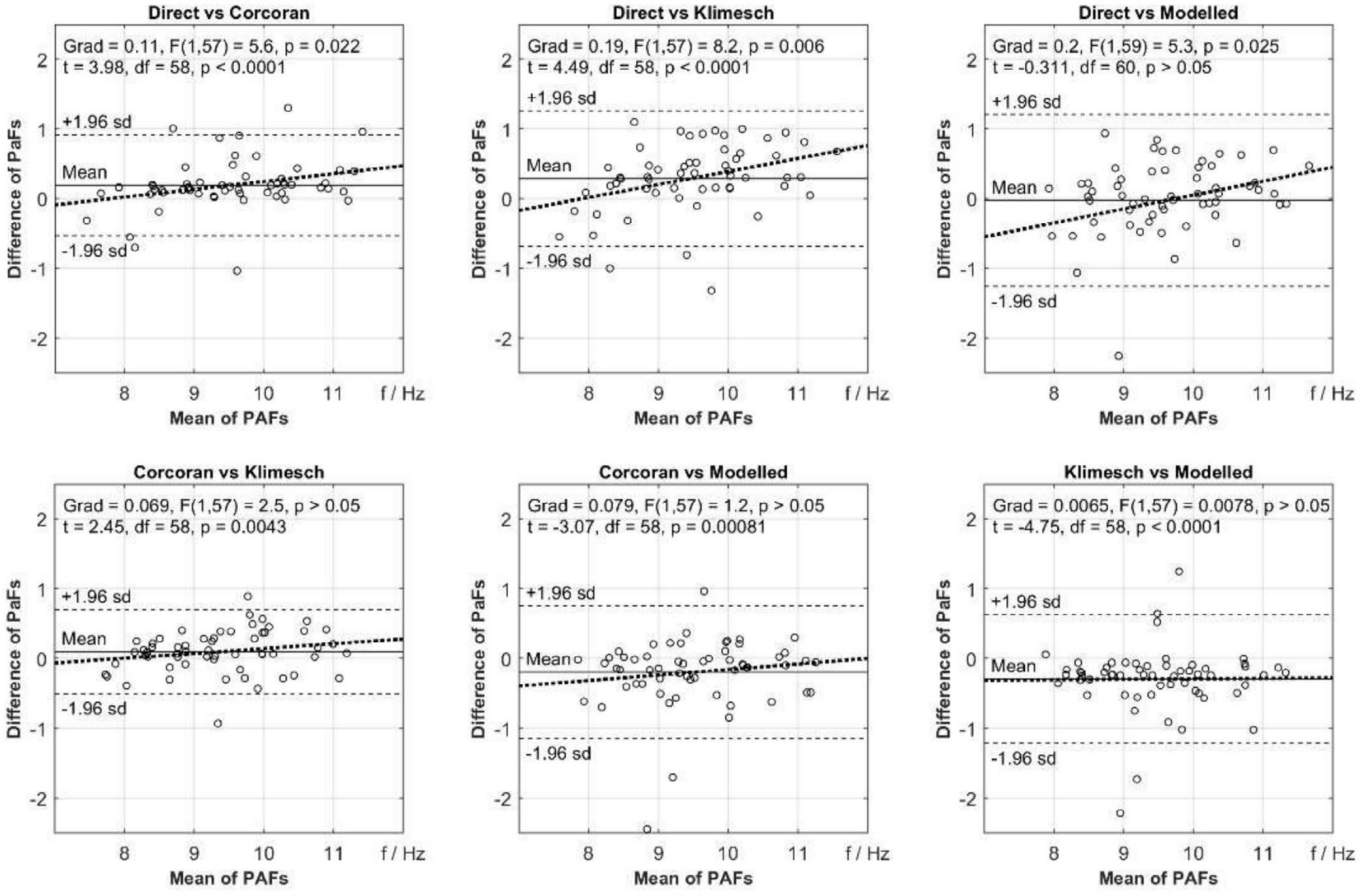
Comparing PAF estimates from the Direct (D‐PAF), Corcoran (C‐PAF), Klimesch (K‐PAF), and Modeled (M‐PAF) methods using Bland–Altman analysis.

A comparison of the four methods of estimating PAF is shown in Figure 3. Using the D‐PAF and M‐PAF methods, estimates of PAF were provided for all participants. Using the C‐PAF and K‐PAF methods, estimates were unavailable for two participants (3%) because there were too few channels with sufficiently prominent alpha peaks. The Bland–Altman analysis reveals that the level of agreement in PAF between methods is relatively poor, with wide 95% limits of agreement (±1.96 SD) and constant error (as shown via the bias of the Mean line) in all except the comparison between D‐PAF and M‐PAF. Indeed, one‐sample t‐tests of the mean values support that the D‐PAF and M‐PAF methods produce estimates of PAF that are not significantly different from each other, but they are significantly higher than C‐PAF, which, in turn, produced significantly higher estimates than K‐PAF (D‐PAF = M‐PAF>C‐PAF>K‐PAF). Additionally, proportional error is present in all comparisons including D‐PAF, suggesting greater bias towards D‐PAF the higher the average PAF. Given the correlational nature of the study, we have also reported the distribution of the PAF variables in Table 2 and a correlation matrix of PAF methods in Table 3.

**TABLE 2:** Distribution of PAF across PAF methods.

	M	SD	Range	Shapiro–Wilk W (*p*)
D‐PAF	9.50	1.04	7.20–11.80	0.99 (> 0.05)
M‐PAF	9.63	0.88	7.85–11.40	0.98 (> 0.05)
C‐PAF	9.42	0.95	7.61–11.20	0.97 (> 0.05)
K‐PAF	9.32	0.89	7.85–11.20	0.97 (> 0.05)

**TABLE 3:** Pearson's *r* correlations between PAF methods.

	D‐PAF	M‐PAF	C‐PAF	K‐PAF
D‐PAF	—			
M‐PAF	0.80*	—		
C‐PAF	0.94*	0.86*	—	
K‐PAF	0.89*	0.86*	0.95*	—

* *p* < 0.001.

### 3.4 Estimating Chronological Age With PAF

**FIGURE 4:**
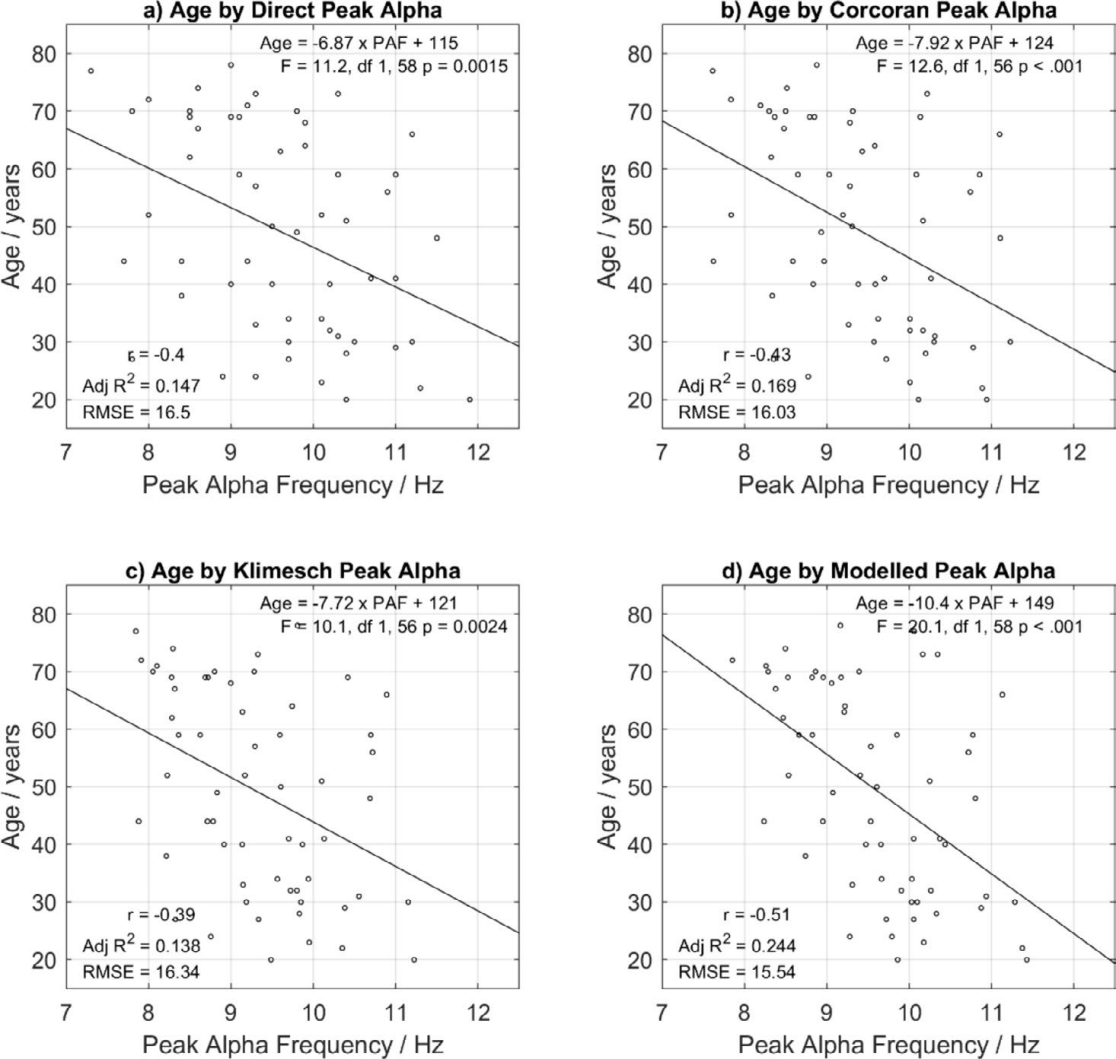
Scattergrams showing the linear relationship between PAF and chronological age. (a) D‐PAF, (b) C‐PAF, (c) K‐PAF, and (d) M‐PAF; *r* = Pearson's correlation coefficient; RMSE = Root Mean Squared Error of Estimation; gradients are in years per Hz.

D‐PAF, M‐PAF, C‐PAF, and K‐PAF were used to look at the relationship between chronological age and PAF, with chronological age regressed onto each estimate of PAF. In each case, PAF proved to be a significant predictor of age (Figure 4), and M‐PAF was the most accurate and strongest predictor of age, descriptively, according to root‐mean‐square error (RMSE) and the correlation coefficient respectively. Using the Glass & Hopkins method to compare the strengths of correlation coefficients between chronological age and PAF age for each method of estimating PAF, Table 4 shows that no method proved significantly stronger than any others (*p* > 0.05). The regression of chronological age on M‐PAF corresponded with a correlation of *r* = −0.51 (or 0.51 on M‐PAF age) and accounted for approximately 24% of the variance in age (RMSE = 15.54). When regressing M‐PAF onto chronological age (RMSE = 0.76), each decade of chronological age was associated with an expected change of 0.25 Hz in PAF $\left(\mathrm{PAF}=-0.025\times \mathrm{Age}+10.8\right)$.

**TABLE 4:** Differences between correlations of chronological age with PAF age for the PAF methods.

	D‐PAF	M‐PAF	C‐PAF	K‐PAF
D‐PAF	—			
M‐PAF	−0.11	—		
C‐PAF	−0.03	0.08	—	
K‐PAF	0.01	0.12	0.04	—

*Note:* The values represent the differences between the respective Pearson's *r* correlation coefficients (e.g., D‐PAF age–M‐PAF age); no method proved significantly stronger than any others (*p* > 0.05).

The M‐PAF method was used to also obtain estimates of peak frequency for theta, beta1, beta2, and gamma, along with the bands' amplitudes and widths. The correlation between the peak frequency, amplitude, and width of each frequency band with chronological age is shown in Table 5. The M‐PAF approach also allowed for the estimation of the amplitude, $A_{0},$ of the $\frac{1}{f^{m}}$ component (i.e., aperiodic 1/*f* slope) of the model, the exponent, *m*, and the offset, k, none of which were significantly correlated with age (*r* = −0.01, −0.16 and 0.02 respectively, *p* > 0.05). There was also no significant correlation between the total amplitude (global) in the 0.1 Hz to 45 Hz frequency range and age (*r* = 0.21, *p* > 0.05).

**TABLE 5:** Correlations of chronological age with peak frequency, amplitude, and width for the five frequency ranges using the M‐PAF method.

	Theta	Alpha	Beta1	Beta2	Gamma
Peak frequency	−0.21	−0.51**	−0.47**	−0.33**	0.08
Amplitude	−0.14	0.01	0.39**	0.28*	−0.10
Width	−0.32*	−0.28*	0.08	0.08	0.33*

* *p* < 0.05.

** *p* < 0.01.

The correlations of chronological age with peak frequency, amplitude, and width seen in multiple frequency bands show that age‐related changes in the EEG power spectrum extend beyond a simple, discrete slowing of the PAF. This finding further supports our examination of the relationship between chronological age and the broad EEG power spectrum of 0.1 to 45 Hz range with PLS.

### 3.5 Estimating Chronological Age With PLS

**FIGURE 5:**
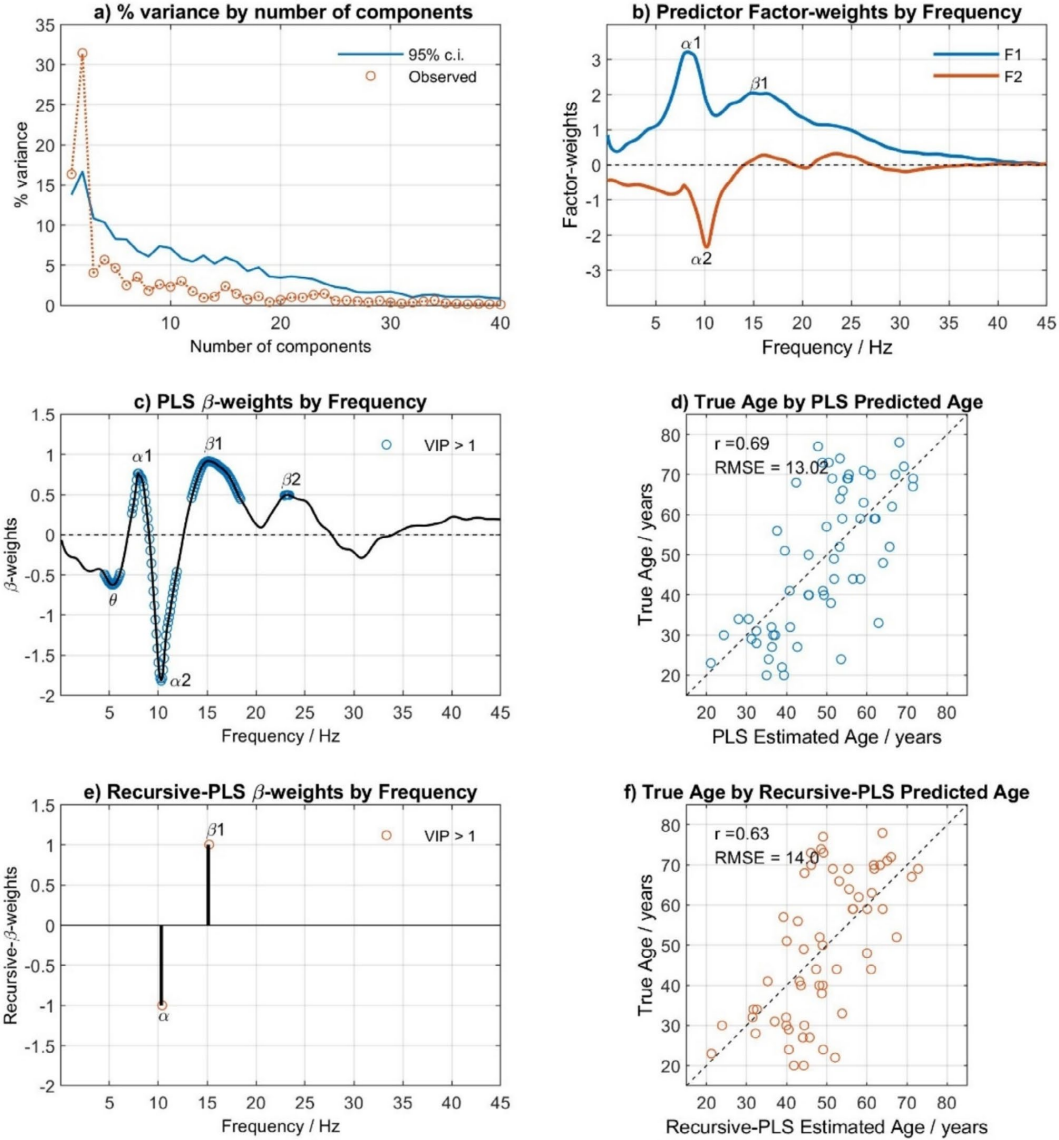
The prediction of chronological age with EEG‐age via PLS analysis of EEG spectra. (a) the percentage variance in chronological age accounted for by the number of components; (b) The factor weights of the two significant factors (i.e., latent variables) by frequency; (c) The two‐dimensional β‐weights by frequency; (d) Chronological age (True Age) by EEG‐age (PLS Estimated Age); (e) The R‐PLS optimal β‐weights by frequency; (f) Chronological age (True Age) by EEG‐age (R‐PLS Estimated Age).

PLS regression was used to look at the relationship between chronological age and EEG age. Chronological age was regressed onto the first SVD‐extracted component of the EEG spectra. Permutation testing revealed that only two factors used to derive β weights were statistically significant (Figure 5a,d). The β weights by frequency are shown in Figure 5b, with frequencies that were significant in predicting age indicated by the Variable Importance in Projection scores (VIP) being > 1 (displayed as a blue circle). Age was most strongly predicted by sections of the EEG spectrum that map onto the low‐alpha and beta frequency ranges (positive weightings) contrasted with frequencies in the theta and high‐alpha ranges (negative weightings). Using these weightings, it was possible to estimate expected chronological age based solely on the EEG spectrum (i.e., EEG‐age) and statistically compare these estimates with true chronological ages. There was a strong positive, significant correlation between PLS EEG age and chronological age (*r* = 0.69, *p* < 0.001; Figure 5c), with the RMSE of 13.02 years showing considerably better accuracy than was achieved using the best PAF estimate to predict chronological age (M‐PAF method; *r* = −0.51, RMSE = 15.54). Adding PLS EEG age as an additional predictor of chronological age, alongside M‐PAF age, significantly improved the fit of a regression model (*R* ^*2*^_*Adjusted*_ = 0.458; *R* ^*2*^_*Change*_ = 0.219, *F*(1,57) = 23.8, *p* < 0.001) and, given the interpretation that PLS correlates of age comprise more information across the broad EEG spectrum than PAF correlates of age, we tested to what extent PLS EEG age remained significantly correlated with age when the PAF is partialled out (*r* = 0.53, *p* < 0.001, controlling for PAF‐M, PAF‐D, PAF‐C, and PAF‐K). Finally, BHR comparing a null model including PLS EEG age with a second model adding in M‐PAF age, in attempts to predict chronological age, reported *BF*_*01*_ = 3.31, providing substantial evidence in favor of *H*_*0*_ (Jeffreys 1961; Wagenmakers et al. 2011) and suggesting EEG age is the important predictor of chronological age.

Identification of the most age‐responsive components of the full EEG power spectrum was determined using R‐PLS. After 14 iterations, a two‐component solution (Figure 5e), which contrasts power at 10.3 Hz (negative weighting) and 15.1 Hz (positive weighting), significantly correlates with chronological age. The RMSE was only a little less good than the full spectrum (Figure 5f; R‐PLS EEG‐age: *r* = 0.62, RMSE = 14, versus PLS EEG‐age: *r* = 0.69, RMSE = 13.02). This suggests that the most important predictor of age is the contrast between alpha and beta1. Using regression to predict chronological age with EEG $\log_{2}\left(\text{amplitude}\right)$ at 10.3 Hz and 15.1 Hz shows a significant relationship (*F*(2,57) = 18.75, *p* < 0.001, *R* ^*2*^_*Adjusted*_ = 0.376, RMSE = 14.0) given by:

(2) $$\mathrm{Age}=68.8-22.5\;A_{10.3\;\mathrm{Hz}}+44.1\;A_{15.1\;\mathrm{Hz}}$$

**FIGURE 6:**
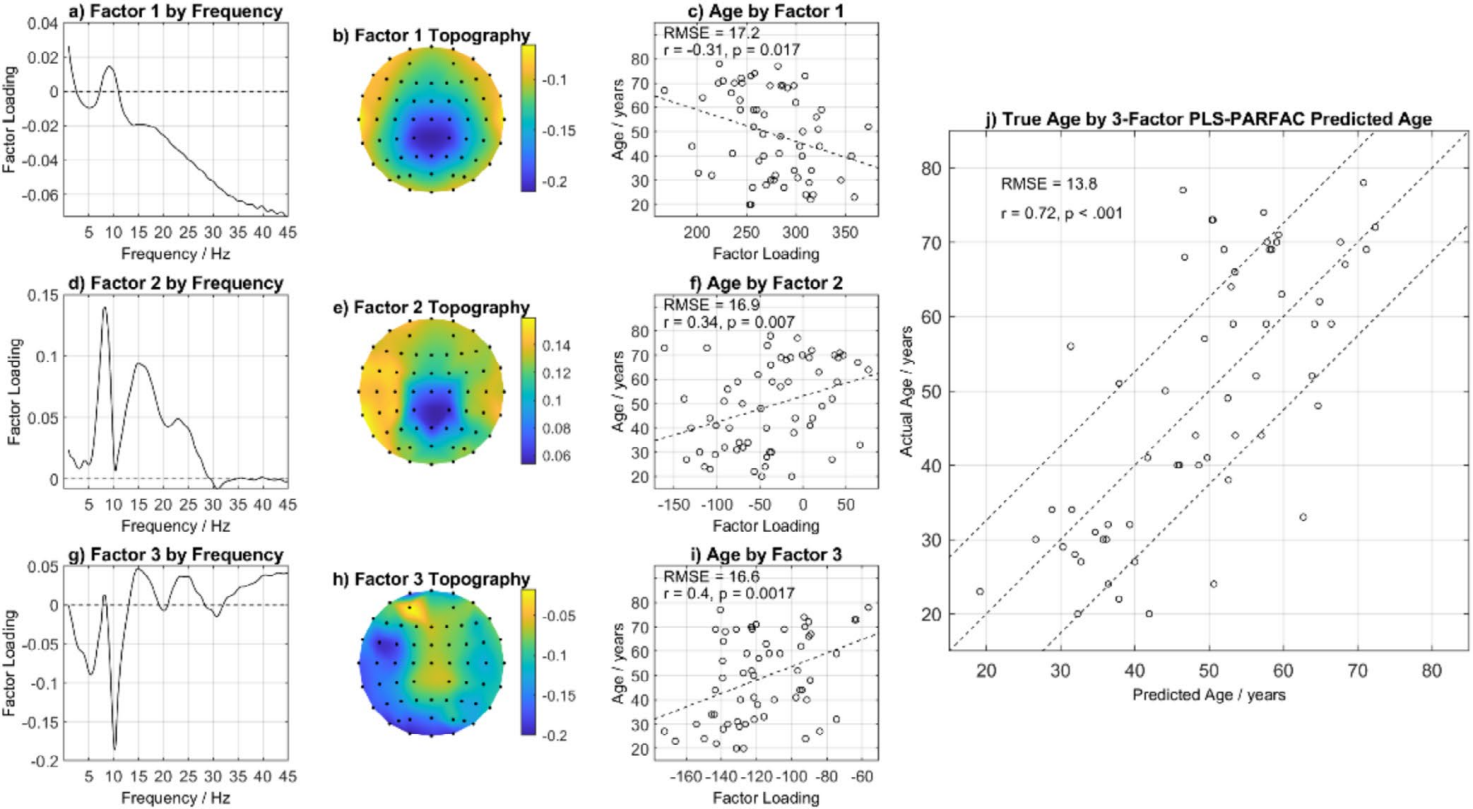
The prediction of chronological age with EEG‐age via M‐PLS analysis of EEG log2(amplitude) spectra. (a, d and g) show the factor loadings by frequency; (b, e and h) show factor weightings by topography; (c, f and i) show the relationships between each factor and chronological age; (j) shows the prediction of chronological age with the three‐factor model of EEG‐age.

M‐PLS (PARAFAC combined with PLS) was implemented to allow for the analysis of a higher‐order array of participants‐by‐channels‐by‐spectra. A model with three factors provided the best fit (Figure 6), with the strongest prediction of age (*R* ^*2*^_*Adjusted*_ = 0.458).

Factor 1 closely resembles the mean EEG‐amplitude spectrum, with the delta and theta frequencies being positively weighted and the other frequencies being negatively weighted, and reflects an underlying, general shift in amplitude from higher to lower frequencies with increasing age (Figure 6a). The negative weightings were most strongly represented at central‐posterior channels (Figure 6b), and this factor was significantly negatively correlated with age (Figure 6c). Factors 2 and 3 are contrast factors focused on different types of transitions in EEG amplitude on either side of 10 Hz and that closely resemble the two components seen in the PLS analysis (Figure 5d). Factor 2 (Figure 6d) showed predominantly positive weightings, including around 15 Hz, that were most strongly represented at peripheral channels and stronger on the left than the right (Figure 6e). Weighting on Factor 2 was significantly, positively correlated with age (Figure 6f). Factor 3 showed a strong negative weighting around 10 Hz, which resided mostly at posterior sites and was also significantly, positively correlated with age (Figure 6g–i). Most importantly, combining the three factors (i.e., M‐PLS EEG‐age) to predict chronological age revealed a strong positive, significant correlation (*r* = 0.72, *p* < 0.001; Figure 6j). This correlation coefficient was comparable to the previous PLS analyses (R‐PLS: *r* = 0.62; PLS: *r* = 0.69). Additionally, the RMSE of 13.8 demonstrates comparable accuracy compared to the previous PLS analyses (R‐PLS: RMSE = 14; PLS: RMSE = 13.02). Given the correlational nature of the study, we have also reported the distribution of the EEG‐age variables in Table 6 and a correlation matrix of PAF and EEG‐age methods in Table 7. Comparing the correlations using the Glass & Hopkins method again, the PLS EEG‐age methods were consistently significantly more strongly correlated with age than the PAF‐age methods, but there was no difference in the magnitudes of the PLS‐age and M‐PLS age correlations (Table 8; *p* > 0.05).

**TABLE 6:** Distribution of EEG‐age across PLS methods.

	M	SD	Range	Shapiro–Wilk W (*p*)
EEG‐age PLS	49.10	12.20	20–73	0.99 (> 0.05)
EEG‐age R‐PLS	49.10	11.30	21–73	0.99 (> 0.05)
EEG‐age M‐PLS	49.10	12.50	17–75	0.99 (> 0.05)

**TABLE 7:** Pearson's *r* correlations between PAF and EEG‐age methods.

	D‐PAF	M‐PAF	C‐PAF	K‐PAF	EEG‐age PLS	EEG‐age R‐PLS	EEG‐age M‐PLS
D‐PAF	—	—	—	—	—	—	—
M‐PAF	0.80**	—	—	—	—	—	—
C‐PAF	0.94**	0.86**	—	—	—	—	—
K‐PAF	0.89**	0.86**	0.95**	—	—	—	—
EEG‐age PLS	−0.42**	−0.62**	−0.45**	−0.42*	—	—	—
EEG‐age R‐PLS	−0.40*	−0.52**	−0.40*	−0.38*	0.79**	—	—
EEG‐age M‐PLS	−0.39*	−0.58**	−0.41*	−0.38*	0.92**	0.90**	—

* *p* < 0.01.

** *p* < 0.001.

**TABLE 8:** Differences between correlations of chronological age with brain age for PAF age and PLS EEG age methods.

	D‐PAF	M‐PAF	C‐PAF	K‐PAF	PLS	R‐PLS	M‐PLS
D‐PAF	—						
M‐PAF	−0.11	—					
C‐PAF	−0.03	0.08	—				
K‐PAF	0.01	0.12	0.04	—			
PLS	−0.28**	−0.18*	−0.25*	−0.29**	—		
R‐PLS	−0.23*	−0.12	−0.20	−0.24*	0.05	—	
M‐PLS	−0.30**	−0.19*	−0.27*	−0.31**	−0.02	−0.07	—

*Note:* The values represent the differences between the respective Pearson's *r* correlation coefficients (e.g., D‐PAF age – M‐PAF age).

* *p* < 0.05.

** *p* < 0.01.

### 3.6 Correlations Between EEG‐Age, NART‐IQ, and Cognitive Performance

Whilst there were no significant differences in the ability to predict chronological age within the PAF and PLS approaches respectively, M‐PAF age and PLS EEG‐age were chosen for the final stages of analysis because they produced the most accurate estimates of age, with the lowest RMSE per approach. It was expected that QMCI would be negatively correlated with age, implicating deleterious age‐related changes such as dedifferentiation and noise. In contrast, neither chronological age nor PLS EEG‐age was significantly correlated with QMCI, but M‐PAF age was significantly correlated with cognitive performance (Table 9). Additionally, a BHR comparing a null model including M‐PAF age with a second model adding in PLS EEG‐age, in attempts to predict QMCI, reported *BF*_*01*_ = 3.51, providing substantial evidence in favor of *H*_*0*_ (Jeffreys 1961; Wagenmakers et al. 2011) and suggesting M‐PAF age is the important predictor of general cognitive functioning. However, a notable inconsistency between EEG‐age and M‐PAF age was that EEG‐age was significantly correlated with NART‐IQ (*r* = 0.43, *p* < 0.001), analogous to the correlation between chronological age and NART‐IQ (*r* = 0.54, *p* < 0.001), but M‐PAF age was not significantly correlated with NART‐IQ (*r* = 0.18, *p* > 0.05). To explore these inconsistencies further, partial correlations were run between ages and cognitive performance accounting for NART‐IQ (Table 9). M‐PAF age remained significantly correlated with cognitive performance, but PLS EEG‐age and chronological age were now also significantly negatively correlated with cognitive performance. Notably, there were no significant differences (*p* > 0.05) in the strength of the correlations for each method once NART‐IQ had been considered (Table 10). Indeed, BHR comparing a null model including M‐PAF age and NART‐IQ with a second model adding in PLS EEG‐age, in attempts to predict QMCI, reported *BF*_*01*_ = 2.56, providing only anecdotal evidence in favor of *H*_*0*_. In summary, these results cannot answer which estimate of brain age is the most strongly correlated with general cognitive functioning.

**TABLE 9:** Correlations of M‐PAF age, PLS EEG age, and chronological age (CA) with QMCI.

	M‐PAF	PLS	CA
QMCI	−0.41**	−0.25	−0.18
QMCI (controlled for NART‐IQ)	−0.45***	−0.33*	−0.29*

* *p* < 0.05.

** *p* < 0.01.

*** *p* < 0.001.

**TABLE 10:** Differences between correlations of age with QMCI for M‐PAF age, PLS EEG age, and chronological age (CA), when accounting for NART‐IQ.

	M‐PAF	PLS	CA
M‐PAF	—		
PLS	−0.12	—	
CA	−0.16	−0.04	—

*Note:* The values represent the differences between the respective Pearson's *r* correlation coefficients (e.g., M‐PAF age – PLS EEG‐age); no method proved significantly stronger than any others (*p* > 0.05).

It was also expected that NART‐IQ would positively correlate with QMCI, implicating protective processes that support cognitive functioning, such as reserve. Indeed, NART‐IQ and QMCI were significantly positively correlated when controlling for chronological age due to the age‐NART‐IQ bias (*r* = 0.26, *p* = 0.047). Additionally, mood and the number of years in formal education were not significantly correlated with any of the EEG measures (*p* > 0.05), which is not overly surprising considering the relatively low levels of depression and small effect sizes when using years in education as a predictor variable. To end, when controlling for both NART‐IQ and chronological age, M‐PAF age remained significantly negatively correlated with cognitive performance (*r* = −0.36, *p* = 0.005) but PLS EEG‐age was not significantly correlated with cognitive performance (*r* = −0.21, *p* > 0.05), which suggests that PAF remains the better estimate of age‐matched, general cognitive functioning.

## 4 Discussion

Our objective was to use the EEG power‐frequency spectrum to estimate chronological age as EEG‐age. First, we compared the well‐established variants of PAF on their ability to estimate age. Irrespective of the peak‐estimation method used, with the methods varying widely in their pre‐specified implementation, PAF was consistently negatively correlated with chronological age, which is consistent with previous evidence that suggests an adult's decline in PAF is a normal brain‐aging phenomenon. We also sought to determine which estimate of PAF (M‐PAF, C‐PAF, K‐PAF, or a variant of N‐PAF) correlated most strongly and provided the most accurate estimate of chronological age. Although there were no statistically significant differences in the strength of their correlations with age, the different methods produced estimates of PAF that varied substantially. This is unsurprising given the very different assumptions and procedures required in each case, but it does underline the lack of consensus as to how best to estimate PAF. Our recommendation is that for the sole purpose of estimating chronological age, the M‐PAF method is to be preferred, primarily because M‐PAF had the lowest expected error (i.e., highest accuracy) out of the PAF‐based estimates of chronological age. Also, due to the use of SVD, M‐PAF did not require mass averaging across electrode channels, and due to the use of AMs, M‐PAF should be able to interrogate and track fine‐grained changes in PAF over time better than other methods. Finally, M‐PAF was successfully adapted to provide estimates of other periodic and aperiodic components of the EEG power spectrum, several of which showed age‐related correlations that suggest a more extensive, complex pattern of age‐related changes than solely a decrease in PAF. We also showed that topographical variation in PAF (i.e., W‐S variance) was relatively unimportant, although we still conclude it would be sensible to use multiple, well‐defined scalp locations that are likely to show clear alpha peaks, reflecting good SNR, to reduce the standard error of the estimated power spectrum.

We then explored the potential of multivariate analyses of the broad EEG spectrum (0.1 to 45 Hz), using PLS and M‐PLS as qualitatively distinct analytical approaches to PAF that have not previously been used to estimate chronological age. Chronological age was estimated with greater accuracy by PLS and M‐PLS than by PAF, and with stronger correlations between chronological age and estimated brain age than reported with PAF and in previous attempts to operationalize an EEG‐age concept (e.g., Al Zoubi et al. 2018; Sun et al. 2019). Additionally, a contrast between alpha and beta1 appeared to be the most age‐responsive aspect of the EEG spectrum, again indicating extensive, complex patterns of age‐related change. The main conclusion thus far is that EEG‐age, estimated from the broad EEG power spectrum, can estimate chronological age with an expected error of between 13 and 14 years and substantially better than well‐established PAF estimates of age. This indicates that EEG‐age is a more comprehensive measure of neural synchronization and, potentially, general integrity. It remains to be seen whether EEG‐age is sufficiently accurate to be useful, though, particularly at the individual level where the baseline variability in EEG metrics is greater, thus making it more difficult to track meaningful change over time than at the group level (Burgess and Gruzelier 1993). However, the correlation coefficient between chronological age and PLS EEG‐age (*r* = 0.69) is the same as the correlation coefficient between scores on the NART and WAIS‐IV (Bright et al. 2018), which, given that the NART is a clinically useful and well‐established tool, suggests our EEG‐age metric may have some utility for tracking general integrity.

One of the several advantages of using the broad EEG power spectrum to estimate EEG‐age is that it removes the need to define frequency band boundaries of interest and eliminates concerns about the number of identifiable peaks. This means that, in contrast to other methods, an estimate of EEG‐age can be obtained in all cases, provided sufficient EEG data has been recorded. Furthermore, using SVD with sophisticated multivariate methods further removes the need to implement mass averaging across electrode channels and allows for all information in the EEG spectra dataset to be used at the same time. PLS appears to be an effective and efficient method of analyzing the power spectrum in the context of aging research, reducing subjective decision‐making during the analysis of EEG data, and providing a more robust estimate of age than the qualitatively distinct PAF‐based approach. It may be preferable to take forward the simpler PLS as opposed to M‐PLS in future studies due to the similarity in overarching conclusions and a lower PLS‐RMSE.

Third, we wanted to examine the relationship of EEG‐age with proxy measures of general cognitive integrity. The relationships between PAF, chronological age, and cognitive functioning were already established, but PLS had not previously been used to estimate EEG‐age before. There was a robust negative correlation between general cognitive performance and the best PAF age estimate, M‐PAF age (i.e., QMCI positively correlated with PAF itself). This is consistent with previous evidence that suggests lower PAF, thus a higher PAF age even with age‐matched samples, likely reflects detrimental changes in neural and cognitive integrity (e.g., dedifferentiation and noise as outlined by the deficit models of aging). PLS EEG‐age and chronological age were both significantly negatively correlated with cognitive performance, as could be expected with an aging sample, only after statistically controlling for NART‐IQ. Furthermore, the strengths of the coefficients were not significantly different from those of the correlation between M‐PAF age and cognitive performance. Taken together, these results suggest that the significant negative correlations between age and general cognitive performance were initially masked by the positive correlation between chronological age and premorbid IQ, which is not overly surprising in this sample of healthy adults who displayed QMCI scores in the normal cognitive functioning range. That is, if the older adults were not scoring in the normal range, their levels of reserve, as proxied by the NART‐IQ, would likely not be so high either. Further supporting this interpretation, NART‐IQ and QMCI were modestly positively correlated after controlling for chronological age, because higher premorbid IQ, thus reserve as outlined by the benefit model of aging, can support better cognitive functioning or at least the maintenance of normal functioning (Cabeza et al. 2018; Stern 2012). The concurrent minor decline in cognitive functioning with chronological age, but not into an MCI range, was initially masking the relationship. In summary, normal age‐related declines in cognitive functioning pull QMCI scores down, whilst an age‐NART‐IQ bias can pull older adults' QMCI scores up concurrently, which is why partial correlation analysis revealed the expected relationships for both age‐QMCI (negative correlation) and NART‐IQ‐QMCI (positive correlation) given the current sample.

Based on the comparisons between PAF and PLS, PLS EEG‐age likely incorporates variance from a wider range of age‐related processes. This is a reasonable assertion given PLS analyses consider a different type of information, amplitude from across the EEG power spectrum rather than an isolated alpha‐band peak frequency, and thus provide a better estimate of chronological age compared to PAF. We know that the EEG power spectrum is a marker of the mass‐synchronized action of cortical neurons (Lopes da Silva 2013) and thus could provide insights into deleterious age‐related conditions from a general, system‐level perspective (Koenig et al. 2020). Therefore, a subset of neural developments that are damaging to general fluid cognitive functioning (e.g., decreased QMCI) might be spotted and tracked via PAF, whereas PLS likely provides a more comprehensive insight into general brain functioning that may reflect a range of both protective (e.g., reserve, benefit model) and deleterious (e.g., dedifferentiation and noise, deficit model) agents, general crystallized and fluid cognition.

The findings of this study should now be confirmed in samples where there is no age –NART –IQ bias and where QMCI scores are not restricted to the normal cognitive functioning range. Indeed, a limitation of this study is that the sample was not representative of the general population in the UK, let alone elsewhere across the globe. The sample was, on average, less ethnically diverse and more intelligent than normative data, and, as discussed already, this difference in intelligence was particularly marked in older adults. Furthermore, this study purposely followed a general approach to the measurement (QMCI and NART) and interpretation (Koenig et al. 2020) of neural and cognitive integrity, but future studies could measure specific subcategories of fluid and crystallized intelligence (e.g., Ociepka et al. 2023). The proxy measures used in this study were not intended to be exhaustive but to provide a starting point in general brain functioning.

Similarly, future studies could provide a systematic, thorough comparison of the different approaches to estimating the PAF and PLS measures. This could include the wide array of different analytical approaches (including approaches not covered here, such as other spectral parameterization algorithms like ‘specparam’; Donoghue et al. 2020), to elucidate specific mechanisms and concrete reasons for the differences observed within and between PAF and PLS EEG‐age. This comparison of technical differences would take us a step closer to confirming whether the PLS EEG‐age estimate outperforms the PAF‐age estimate simply because it uses more data from the EEG spectrum. It might be, for example, that the difference is due to the method of spectral analysis used. That said, our current analyses indicate that the method of spectral analysis, smoothing, and agglomeration made relatively little difference to the PAF‐age correlation, which would suggest that the PLS‐age correlation is stronger than the PAF‐age correlation because PLS uses more information from the EEG spectrum. In summary, it will be necessary to establish good, large‐scale normative data that makes it possible to estimate the EEG age of individuals across the entire human lifespan, not just adulthood, with high reliability, validity, and precision. This would facilitate the identification of individuals with unusual discrepancies between their EEG age and the normative EEG age for their chronological age and condition.

To determine the utility of such a discrepancy, researchers could calculate a cognitive‐discrepancy score, such as NART‐IQ (expected) compared to WAIS‐IQ (current; e.g., Nelson 1982; Nelson and Willison 1991), and interpret that alongside an age‐discrepancy score, such as chronological age (expected) compared to EEG‐age (current; e.g., Al Zoubi et al. 2018; Wackermann and Matoušek 1998). However, researchers would also need to determine whether an age‐discrepancy score is useful at an early stage of disease and reflects specific problems (e.g., acting as a reliable diagnostic test for Alzheimer's disease) or a general pre‐condition brain state that is indicative of a range of potential pathologies. Therefore, it will be necessary to recruit participants with specific age‐related conditions, such as subjective cognitive impairment (SCI), MCI, and established dementia, of varying etiologies, to determine the practical utility of an age‐discrepancy score. A suitably a priori‐powered longitudinal study would allow for a robust examination of EEG‐age over time and could allow for identifying problematic conditions early on. SCI has been hard to define and detect with other objective measures as it may be driven by anxiety/affect rather than a neural condition, but could be an early precursor to future, clinically‐observable decline (Reisberg et al. 2010; Stewart 2012; Yue et al. 2021). MCI also remains difficult to detect but is a proven precursor stage to Alzheimer's disease (AD), with disease progression estimated to occur in 15%–41% of MCI cases (Davis et al. 2018; Gauthier et al. 2006; Geslani et al. 2005; Ritchie 2004).

Early detection of dementia or a precursor stage is critical because around 40% of dementia cases worldwide are accounted for by 12 modifiable risk factors across the lifespan (Livingstone et al. 2020), and many of these risk factors also account for the development of MCI (Apostolo et al. 2016) and reversion of MCI back to normal aging (Sanz‐Blasco et al. 2022). In other words, dementia is not inevitable, and a general picture of a person's neural and cognitive integrity over time could allow for proactive intervention, including relatively simple lifestyle changes that may prevent decline. Additionally, Lecanemab and Donanemab are drugs that may slow the speed at which AD progresses, albeit to a modest extent and with side effects (Sims et al. 2023; van Dyck et al. 2023), but to be effective, these drugs need to be ingested at the optimal point in time, which is only possible with earlier detection. While the idea of using EEG‐age as an early‐detection biomarker is not new (e.g., Al Zoubi et al. 2018; Sun et al. 2019), our approach to estimating EEG‐age already has advantages over previous attempts. We are proposing a specific EEG measure that is simple, quick, relatively cheap, and safe to collect. It requires no behavioral input or active effort from the participant and is a relatively objective and stable measurement. It also reduces several subjective analytical decisions on the part of the researcher. Additionally, our study has incorporated a more age‐balanced sample of exclusively healthy adults compared to previous approaches to examining EEG‐age (e.g., Matoušek and Petersén 1973; Wackermann and Matoušek 1998). In summary, although our EEG‐age metric has advantages over many of the traditional neuroimaging methodologies and neuropsychological measures, there is a long way to go before it can be used in a clinical setting.

Looking to future research, EEG‐age may be most useful when used in conjunction with other indicators in an optimized form of diagnostic triangulation (e.g., alongside other neuroimaging and physiological markers, including MEG, MRI, blood and plasma tests, DNA‐mapping, as well as neuropsychological and cognitive tasks, self‐report questionnaires, and interviews with family members). Combinations of measures can already predict conversion from normal aging to MCI to AD with good sensitivity and specificity (Apostolo et al. 2016; Dauwels et al. 2010; Huang et al. 2000; Poil et al. 2013; Snaedal et al. 2012; Weiner et al. 2022), so we posit that incorporating an optimized version of our EEG‐age metric, comprising even simpler, accessible data collection (e.g., fewer EEG electrodes) and robust, reliable analysis (e.g., an automated procedure), would augment these scores and enable detection at an earlier stage (Koenig et al. 2020). As part of optimizing EEG‐age, it would also be worthwhile to examine the 1/f gradient in greater detail and incorporate the most extreme ends of the EEG spectrum, particularly low frequencies (< 0.01 Hz), as this would likely improve the ability of EEG‐age to estimate chronological age.

To conclude, our vision for the future is a reliable, valid, and precise estimate of EEG‐age for comparison with chronological age. Our objective in the current study was to kickstart that vision, and we have reported, for the first time, that chronological age was estimated with greater accuracy by a multivariate PLS analysis of the broad EEG power‐frequency spectrum (0.1–45 Hz) than by PAF. While a restricted sample of healthy adults with a chronological age –NART‐IQ bias has limited the scope of the current conclusions, and a thorough comparison of the technical differences within and between the calculations of PAF‐ and EEG‐ages would be valuable, we propose that EEG‐age could be refined into a biomarker for neural and cognitive integrity. A discrepancy between EEG‐age and chronological age could prove clinically informative by implicating protective or deleterious age‐related change earlier than previously possible.

## Author Contributions

**Thomas M. James:** data curation, formal analysis, investigation, methodology, project administration, resources, software, validation, visualization, writing – original draft, writing – review and editing. **Adrian P. Burgess:** conceptualization, data curation, formal analysis, funding acquisition, methodology, resources, software, supervision, validation, visualization, writing – original draft, writing – review and editing.

## Conflicts of Interest

The authors declare no conflicts of interest.

## Data Availability

Data are available at https://osf.io/46pzm; a preprint is available at PsyArXiv (https://psyarxiv.com/4sfdm).
